# Tail properties and approximate distribution and expansion for extreme of LGMD

**DOI:** 10.1186/s13660-017-1316-0

**Published:** 2017-02-15

**Authors:** Jianwen Huang, Jianjun Wang, Guowang Luo, Jun He

**Affiliations:** 1 School of Mathematics and Statistics, Southwest University, Chongqing, 400715 China; 2 School of Mathematics and Computational Science, Zunyi Normal College, Zunyi, 563002 China

**Keywords:** 60G70, 60F05, limit distribution, logarithmic generalized Maxwell distribution, Mills’ ratio, maximum, tail properties

## Abstract

We introduce logarithmic generalized Maxwell distribution motivated by Vodă (Math. Rep. 11:171-179, 2009), which is an extension of the generalized Maxwell distribution. Some interesting properties of this distribution are studied and the asymptotic distribution of the partial maximum of an i.i.d. sequence from the logarithmic generalized Maxwell distribution is gained. The expansion of the limit distribution from the normalized maxima is established under the optimal norming constants, which shows the rate of convergence of the distribution for normalized maximum to the extreme limit.

## Introduction

The generalized Maxwell distribution (GMD for short), a generalization of ordinary Maxwell (or classical Maxwell) distribution, was proposed by Vodă [1]. With the rapid development of economy and science and technology, some of the existing distribution functions cannot meet the needs of research. For example, for some skewed data, it is appropriate to describe and fit them only by using some logarithmic models. Therefore, the recent development of some new distribution functions and the study of logarithmic case of the distribution functions have become hot issues in the statistical field. For more details, please refer to [2–8]. In this paper, we define the logarithmic generalized Maxwell distribution (for brevity LGMD), which is a natural prolongation of the generalized Maxwell distribution. In addition to the previously mentioned, one motivation of thinking of LGMD is to obtain more efficient results as parameter estimators when random models were supposed with the LGMD error terms instead of normal ones. Other aspects, like compressive sensing, we hope the LGMD could be used to model impulsive noise [9].

The GMD has a variety of applications in statistics, physics, and chemistry. The probability density function (pdf) and the cumulative distribution function (cdf) of the GMD with the parameter $k>0$ are respectively,

$$g_{k}(x)=\frac{k}{2^{k/2}\sigma^{2+1/k}\Gamma(1+k/2)}x^{2k}\exp \biggl(- \frac{x^{2k}}{2\sigma^{2}} \biggr) $$

and

$$G_{k}(x)= \int^{x}_{-\infty}g_{k}(t)\, \mathrm{d}t $$

for $x\in R$, where *σ* is a positive constant and $\Gamma(\cdot)$ is the gamma function.

Mills [10] gave a well-known inequality and Mills’ ratio conclusion for the standard Gauss cdf $\Phi(x)$ with respect to its pdf $\phi(x)$ as follows:

1.1 $$ x^{-1}\bigl(1+x^{-2}\bigr)^{-1} \phi(x)< \Phi(-x)< x^{-1}\phi(x) $$

for $x>0$, and

1.2 $$ \frac{\Phi(-x)}{\phi(x)}\sim\frac{1}{x}, $$

as $x\rightarrow\infty$.

Peng *et al.* [11] extended the Mills results to the case of the general error distribution:

1.3 $$ \frac{2\lambda^{v}}{v}x^{1-v} \biggl(1+\frac{2(v-1)\lambda ^{v}}{v}x^{-v} \biggr)^{-1}< \frac{T_{v}(-x)}{t_{v}(x)}< \frac{2\lambda^{v}}{v}x^{1-v} $$

for $v>1$ and $x>0$, and for $v>0$

1.4 $$ \frac{T_{v}(-x)}{t_{v}(x)}\sim\frac{2\lambda^{v}}{v}x^{1-v}, $$

as $x\rightarrow\infty$, where $\lambda= [\frac{2^{-2/v}\Gamma(1/v)}{\Gamma(3/v)} ]^{1/2}$, and $T_{v}(x)$ is the general error cdf with pdf $t_{v}(x)$. Huang and Chen [12] investigated similar results of GMD, *viz.*,

1.5 $$ \frac{\sigma^{2}}{k}x^{1-2k}< \frac{1-G_{k}(x)}{g_{k}(x)}< \frac{\sigma ^{2}}{k}x^{1-2k} \biggl(1+ \biggl(\frac{\sigma^{2}}{k}x^{2k}-1 \biggr)^{-1} \biggr) $$

for $k>1/2$, $\sigma>0$ and $x>0$, and for $k>0$,

1.6 $$ \frac{1-G_{k}(x)}{g_{k}(x)}\sim\frac{\sigma^{2}}{k}x^{1-2k}, $$

as $x\rightarrow\infty$. The above-mentioned Mill type inequalities such as (1.1), (1.3), and (1.5) and Mills’ type ratios such as (1.2), (1.4), and (1.6) play an important role in considering some tail behavior and extremes of economic and financial data.

The present paper is to derive the Mills’ inequality, Mills’ ratio, and the distributional tail expression for the LGMD. As an important application, the asymptotic distribution of the partial maximum of i.i.d. variables with common LGMD is investigated. As another significant application, with appropriate normalized constants, the distributional expansion of the normalized maxima from LGMD is obtained. Moreover, we indicate that rate of convergence of the distribution of normalized maxima to corresponding extreme value limit is of the order of $O (1/(\log n)^{1-1/(2k)} )$.

First of all, we provide the definition of LGMD.

### Definition 1.1

Set *X* stand for a random variable which obeys the GMD. Set $Y=\exp(X)$. Then *Y* is termed obeying the LGMD, denoted by $Y\sim \operatorname{LGMD}(k)$ with parameter $k>0$.

Easily check that the pdf is

$$f_{k}(x)=\frac{kx^{-1}}{2^{k/2}\sigma^{2+1/k}\Gamma(1+k/2)}(\log x)^{2k}\exp \biggl(- \frac{(\log x)^{2k}}{2\sigma^{2}} \biggr) $$

for $x>0$, where parameter $k>0$, and *σ* is a positive constant. Suppose that

$$F_{k}(x)= \int^{x}_{0}f_{k}(t)\, \mathrm{d}t $$

for $x>0$. Observe that the LGMD decreases to the logarithmic Maxwell distribution when $k=1$.

The rest of the article is organized as follows. In Section 2, we derive some interesting results including Mills-type ratios and tail behaviors of LGMD. In Section 3, we discuss the asymptotic distribution of normalized maxima of i.i.d. random variables following the LGMD and the suitable norming constants. We generalize the result to the case of a finite blending of LGMDs. In Section 4, we establish the asymptotic expansion of the distribution of the normalized maximum from LGMD under optimal choice of norming constants. As a byproduct, we obtain the convergence speed of the distribution of the normalized partial maxima to its limit.

## Mills’ ratio and tail properties of LGMD

In this part, we obtain some significant results including Mills’ inequality, Mills’ ratio of LGMD.

As to LGMD and GMD, observe that $1-G_{k}(\log x)=1-F_{k}(x)$ and

$$\frac{1-G_{k}(\log x)}{x^{-1}g_{k}(\log x)}=\frac{1-F_{k}(x)}{f_{k}(x)}. $$

Hence, by Lemma 2.2 and Theorem 2.1 in Huang and Chen [12], the two results below follow.

### Theorem 2.1

*Suppose that* $F_{k}$ *and* $f_{k}$ *respectively represent the cdf and pdf of LGMD with parameter* $k>1/2$. *We have the inequality below*, *for all* $x>1$,

2.1 $$ \frac{\sigma^{2}}{k}x(\log x)^{1-2k}< \frac{1-F_{k}(x)}{f_{k}(x)}< \frac{\sigma^{2}}{k}x(\log x)^{1-2k} \biggl(1+ \biggl(\frac{\sigma^{2}}{k}( \log x)^{2k}-1 \biggr)^{-1} \biggr), $$

*where* *σ* *is a positive constant*.

### Corollary 2.1

*For fixed* $k>0$, *as* $x\rightarrow\infty$, *we have*

2.2 $$ \frac{1-F_{k}(x)}{f_{k}(x)}\sim\frac{\sigma^{2}}{k}x(\log x)^{1-2k}. $$

### Remark 2.1

Since the $\operatorname{LGMD}(k)$ are reduced to the logarithmic Maxwell distribution as $k=1$, so by Theorem 2.1 and Corollary 2.1, we derive the Mill’s inequality and Mills’ ratio of the logarithmic Maxwell distribution, *viz.*,

$$\sigma^{2}x(\log x)^{-1}f_{1}(x)< 1-F_{1}(x)< \sigma^{2}x(\log x)^{-1}\bigl(1+\bigl(\sigma^{2}( \log x)^{2}-1\bigr)^{-1}\bigr)f_{1}(x) $$

for $x>1$, and

$$\frac{1-F_{1}(x)}{f_{1}(x)}\sim\frac{\sigma^{2}x}{\log x}, $$

as $x\rightarrow\infty$.

### Remark 2.2

For $k>1/2$, Corollary 2.1 gives $F_{k}\in D(\Lambda)$, *i.e.*, there are norming constants $\alpha_{n}>0$ and $\beta_{n}\in\mathbb{R}$ which ensure $F^{n}_{k}(\alpha_{n}x+\beta_{n})$ converges to $\exp(-\exp(-x))$, as $n\rightarrow\infty$. Since

$$\frac{(\mathrm{d}/\mathrm{d}x)f_{k}(x)}{f_{k}(x)}=-\frac{1}{x} \biggl(1-\frac{2k}{\log x}+ \frac{k}{\sigma^{2}}(\log x)^{2k-1} \biggr), $$

by Corollary 2.1, we obtain

$$\frac{1-F_{k}(x)}{f_{k}(x)}\frac{(\mathrm{d}/\mathrm{d}x)f_{k}(x)}{f_{k}(x)}\rightarrow-1, $$

as $x\rightarrow\infty$. Hence, applying Proposition 1.18 in Resnick [13], we obtain $F_{k}\in D(\Lambda)$. As to how to choose the norming constants $\alpha_{n}$ and $\beta_{n}$ will be explored by Theorem 3.2.

Finner *et al.* [14] investigated the asymptotic property of the ratio of the Student *t* and Gauss distributions as the degrees of freedom $u=u(x)$ satisfies

2.3 $$ \lim_{x\rightarrow\infty}\frac{x^{4}}{u}=\beta\in[0,\infty). $$

The main motivation of the work is to consider the false discovery rate in multiple testing problems with large numbers of hypotheses and extremely small critical values for the smallest ordered *p* value; for details, see Finner *et al.* [15]. In the following, we investigate the asymptotic property of the ratio of pdfs and the ratio of the tails of the LGMD and the logarithmic Maxwell distribution. Firstly, we think over the situation of $k\rightarrow1$. Secondly, we think over the situation of $x\rightarrow\infty$ for fixed *k*.

### Theorem 2.2

*For* $k>0$, *let* $x=x(k)$ *be such that*

2.4 $$ k-1=\frac{\gamma}{2(\log x)^{2}\log\log x} $$

*for some* $\gamma\in\mathbb{R}$. *We obtain*

2.5 $$ \lim_{k\rightarrow1}\frac{f_{1}(x)}{f_{k}(x)}=\exp \biggl( \frac{\gamma }{2\sigma^{2}} \biggr) $$

*and*

2.6 $$ \lim_{k\rightarrow1}\frac{1-F_{1}(x)}{1-F_{k}(x)}=\exp \biggl( \frac {\gamma}{2\sigma^{2}} \biggr). $$

### Proof

Observe that $\frac{2^{(k+1)/2}\sigma^{2+1/k}\Gamma(1+k/2)}{k\sigma^{3}\pi ^{1/2}}\rightarrow1$ as $k\rightarrow1$, therefore

$$\begin{aligned} \lim_{k\rightarrow1}\frac{f_{1}(x)}{f_{k}(x)}&=\lim_{k\rightarrow1}( \log x)^{2-2k}\exp \biggl(\frac{(\log x)^{2k}}{2\sigma^{2}}-\frac{(\log x)^{2}}{2\sigma^{2}} \biggr) \\ &=\lim_{k\rightarrow1}\exp \biggl(\frac{(\log x)^{2}}{2\sigma^{2}} \bigl((\log x)^{2k-2}-1 \bigr) \biggr) \\ &=\lim_{k\rightarrow1}\exp \biggl(\frac{(\log x)^{2}}{2\sigma^{2}}\bigl(\exp \bigl((2k-2)\log\log x\bigr)-1\bigr) \biggr) \\ &=\lim_{k\rightarrow1}\exp \biggl(\frac{(\log x)^{2}}{2\sigma^{2}} \biggl(\exp \biggl( \frac{\gamma}{(\log x)^{2}} \biggr)-1 \biggr) \biggr) \\ &=\exp \biggl(\frac{\gamma}{2\sigma^{2}} \biggr). \end{aligned}$$

By (2.4), it is easy to check that $x\rightarrow\infty$ as $k\rightarrow1$. Again applying (2.4), we have

2.7 $$\begin{aligned} (\log x)^{2-2k}&=\exp\bigl(2(1-k)\log\log x\bigr) \\ &=\exp \biggl(\frac{\gamma}{2(\log x)^{2}} \biggr)\rightarrow1,\quad \mbox{as } k \rightarrow1. \end{aligned}$$

Combining (2.7), Corollary 2.1, Remark 2.1, and (2.5), representation (2.6) can be derived. □

### Theorem 2.3

*For fixed* *k*, *we have*

2.8 $$ \frac{f_{1}(x)}{f_{k}(\exp((\log x)^{1/k}))}=\frac{2^{(k+1)/2}\Gamma(1+k/2)\exp((\log x)^{1/k})}{\pi^{1/2}k\sigma^{1-1/k}x} $$

*and*

2.9 $$ \lim_{x\rightarrow\infty}\frac{(\log x)^{1/k-1}(1-F_{1}(x))}{1-F_{k}(\exp((\log x)^{1/k}))}=\frac{2^{(k+1)/2}\Gamma(1+k/2)}{\pi^{1/2}\sigma^{1-1/k}}. $$

### Proof

It is easy to verify (2.8) by fundamental calculation. By Corollary 2.1, Remark 2.1, and (2.8), we have

$$\begin{aligned}& \lim_{x\rightarrow\infty}\frac{(\log x)^{1/k-1}(1-F_{1}(x))}{1-F_{k}(\exp((\log x)^{1/k}))} \\& \quad = \lim_{x\rightarrow\infty}\frac{kx}{\exp((\log x)^{1/k})}\frac{f_{1}(x)}{f_{k}(\exp((\log x)^{1/k}))} \\& \quad = \frac{2^{(k+1)/2}\Gamma(1+k/2)}{\pi^{1/2}\sigma^{1-1/k}}. \end{aligned}$$

Hence (2.9) follows. □

## Limiting distribution of the maxima

By applying Corollary 2.1, we could establish the distributional tail representation for the LGMD.

### Theorem 3.1

*Under the conditions of Theorem* 2.1, *we have*

$$1-F_{k}(x)=c(x)\exp \biggl(- \int^{x}_{e}\frac{g(t)}{f(t)} \, \mathrm {dt} \biggr) $$

*for large enough* *x*, *where*

$$c(x)=\frac{1}{2^{k/2}\sigma^{1/k}\Gamma(1+k/2)}\exp \bigl(-1/\bigl(2\sigma^{2}\bigr) \bigr) \bigl(1+\theta_{1}(x)\bigr) $$

*and*

$$f(t)=\frac{\sigma^{2}}{k}t(\log t)^{1-2k},\qquad g(t)=1- \frac{\sigma ^{2}}{k}(\log t)^{-2k}, $$

*where* $\theta_{1}(x)\rightarrow0$ *as* $x\rightarrow\infty$.

### Proof

For large enough *x*, by Corollary 2.1, we have

$$\begin{aligned} 1-F_{k}(x) =&\frac{\sigma^{2}}{k}(\log x)^{1-2k}xf_{k}(x) \bigl(1+\theta_{1}(x)\bigr) \\ =&\frac{1}{2^{k/2}\sigma^{1/k}\Gamma(1+k/2)}\exp \biggl(\log \log x-\frac{(\log x)^{2k}}{2\sigma^{2}} \biggr) \bigl(1+ \theta_{1}(x)\bigr) \\ =&\frac{1}{2^{k/2}\sigma^{1/k}\Gamma(1+k/2)}\exp \biggl(-\frac{1}{2\sigma^{2}} \biggr)\exp \biggl(- \int^{x}_{e} \biggl(\frac {k(\log t)^{2k-1}}{\sigma^{2}t}- \frac{1}{t\log t} \biggr) \, \mathrm{dt} \biggr) \\ &{}\times \bigl(1+\theta_{1}(x) \bigr) \\ =&\frac{1}{2^{k/2}\sigma^{1/k}\Gamma(1+k/2)}\exp \biggl(-\frac{1}{2\sigma^{2}} \biggr)\exp \biggl(- \int^{x}_{e}\frac {1-k^{-1}\sigma^{2}(\log t)^{-2k}}{k^{-1}\sigma^{2}t(\log t)^{1-2k}}\, \mathrm{dt} \biggr) \\ &{}\times\bigl(1+\theta_{1}(x)\bigr) \\ =&c(x)\exp \biggl(- \int^{x}_{e}\frac{g(t)}{f(t)}\, \mathrm {dt} \biggr), \end{aligned}$$

where $\theta_{1}(x)\rightarrow0$ as $x\rightarrow\infty$. The desired result follows. □

### Remark 3.1

As $\lim_{t\rightarrow\infty}g(t)=1$, $f(t)>0$ on $[1,\infty)$ is absolutely continuous function and $\lim_{t\rightarrow\infty}f'(t)=0$ in Theorem 3.1, an application of Theorem 3.1 and Corollary 1.7 in Resnick [13] shows $F_{k}\in D(\Lambda)$, and the norming constants $a_{n}$ and $b_{n}$ can be chosen by

3.1 $$ \frac{1}{1-F_{k}(b_{n})}=n, \qquad a_{n}=f(b_{n}) $$

such that

3.2 $$ \lim_{n\rightarrow\infty}F^{n}_{k}(a_{n}x+b_{n})= \Lambda(x), $$

where $D(\Lambda)$ denotes the domain of attraction $\Lambda(x)=\exp(-\exp(-x))$.

Here we establish the asymptotic distribution of the normalized maximum of a sequence of i.i.d. random variables following LGMD. Remark 2.2 and Theorem 3.1 showed that the distribution of partial maximum converges to $\Lambda(x)$. So, the following task is to look for the associated suitable norming constants.

### Theorem 3.2

*Suppose that* $\{X_{n},n\geq1\}$ *be an i*.*i*.*d*. *sequence from the LGMD with* $k>1/2$. *Let* $M_{n}=\max\{X_{1},X_{2},\ldots,X_{n}\}$. *We have*

$$\lim_{n\rightarrow\infty}P(M_{n}\leq\alpha_{n}x+ \beta_{n})=\exp\bigl(-\exp(-x)\bigr), $$

*where*

$$ \alpha_{n} =\frac{\sigma^{2}\exp (2^{1/(2k)}\sigma^{1/k}(\log n)^{1/(2k)} ) (1+\frac{\sigma^{1/k}(\log n)^{1/(2k)-1}}{2^{2-1/(2k)}k^{2}}(\log\log n-(k^{2}-1)\log2-2k\log\Gamma(1+k/2)) )}{ k (2^{1/(2k)}\sigma^{1/k}(\log n)^{1/(2k)}+\log (1+\frac{\sigma^{1/k}(\log n)^{1/(2k)-1}}{2^{2-1/(2k)}k^{2}}(\log\log n-(k^{2}-1)\log2-2k\log\Gamma(1+k/2)) ) )^{2k-1}} $$

*and*

$$\begin{aligned} \beta_{n} =&\exp \bigl(2^{1/(2k)}\sigma^{1/k}(\log n)^{1/(2k)} \bigr) \biggl(1+\frac{\sigma^{1/k}(\log n)^{1/(2k)-1}}{2^{2-1/(2k)}k^{2}} \bigl(\log\log n- \bigl(k^{2}-1\bigr)\log2 \\ &{}-2k\log\Gamma(1+k/2) \bigr) \biggr). \end{aligned}$$

### Proof

Since $F_{k}\in D(\Lambda)$, there must be norming constants $a_{n}>0$ and $b_{n}\in\mathbb{R}$ which make sure that $\lim_{n\rightarrow\infty}P((M_{n}-b_{n})/a_{n}\leq x)=\exp(-\exp(-x))$. By Proposition 1.1 in Resnick [13] and Theorem 3.1, we can make choice of the norming constants $a_{n}$ and $b_{n}$ satisfying the equations: $b_{n}=(1/(1-F_{k}))^{\leftarrow}(n)$ and $a_{n}=f(b_{n})$. Note that $F_{k}(x)$ is continuous, then $1-F_{k}(b_{n})=n^{-1}$. By Corollary 2.1, we have

$$nk^{-1}\sigma^{2}(\log b_{n})^{1-2k}b_{n}f_{k}(b_{n}) \rightarrow1, $$

as $n\rightarrow\infty$, *viz.*,

$$n2^{-\frac{k}{2}}\sigma^{-\frac{1}{k}}\Gamma^{-1} \biggl(1+ \frac {k}{2} \biggr)\log b_{n}\exp \biggl(-\frac{(\log b_{n})^{2k}}{2\sigma ^{2}} \biggr)\rightarrow1, $$

as $n\rightarrow\infty$, and so

3.3 $$ \log n-\frac{k}{2}\log2-\frac{1}{k}\log\sigma-\log \Gamma \biggl(1+\frac{k}{2} \biggr)+\log\log b_{n}- \frac{(\log b_{n})^{2k}}{2\sigma^{2}}\rightarrow0, $$

as $n\rightarrow\infty$, from which one deduces

$$\frac{(\log b_{n})^{2k}}{2\sigma^{2}\log n}\rightarrow1, $$

as $n\rightarrow\infty$, thus

$$2k\log\log b_{n}-\log2-2\log\sigma-\log\log n\rightarrow0, $$

as $n\rightarrow\infty$, hence

$$\log\log b_{n}=\frac{1}{2k}(\log2+2\log\sigma+\log\log n)+o(1). $$

Putting the equality above into (3.3), we have

$$(\log b_{n})^{2k}=2\sigma^{2} \biggl(\log n+ \frac{1}{2k}\log\log n-\frac {k^{2}-1}{2k}\log2-\log\Gamma \biggl(1+ \frac{k}{2} \biggr) \biggr)+o(1), $$

from which one induces that

$$\log b_{n}=2^{\frac{1}{2k}}\sigma^{\frac{1}{k}}(\log n)^{\frac {1}{2k}} \biggl(1+\frac{\log\log n-(k^{2}-1)\log2-2k\log\Gamma (1+\frac{k}{2})}{2^{2}k^{2}\log n}+o \bigl((\log n)^{-1} \bigr) \biggr), $$

therefore

$$\begin{aligned} b_{n} =&\exp \bigl(2^{\frac{1}{2k}}\sigma^{\frac{1}{k}}(\log n)^{\frac{1}{2k}} \bigr) \biggl(1+\frac{\sigma^{\frac{1}{k}}(\log n)^{\frac{1}{2k}-1}}{2^{2-\frac{1}{2k}}k^{2}} \biggl(\log\log n- \bigl(k^{2}-1\bigr)\log2 \\ &{}-2k\log\Gamma\biggl(1+\frac{k}{2}\biggr) \biggr)+o \bigl((\log n)^{\frac{1}{2k}-1} \bigr) \biggr) \\ =&\beta_{n}+o \bigl((\log n)^{\frac{1}{2k}-1}\exp \bigl(2^{\frac{1}{2k}} \sigma^{\frac {1}{k}}(\log n)^{\frac{1}{2k}} \bigr) \bigr), \end{aligned}$$

where

$$\begin{aligned} \beta_{n} =&\exp \bigl(2^{1/(2k)}\sigma^{1/k}(\log n)^{1/(2k)} \bigr) \biggl(1+\frac{\sigma^{1/k}(\log n)^{1/(2k)-1}}{2^{2-1/(2k)}k^{2}}\bigl(\log\log n- \bigl(k^{2}-1\bigr)\log2 \\ &{}-2k\log\Gamma(1+k/2)\bigr) \biggr). \end{aligned}$$

Hence, we have

$$\begin{aligned} \alpha_{n}&=f(\beta_{n}) \\ &=\frac{\sigma^{2}\exp(2^{1/(2k)}\sigma^{1/k}(\log n)^{1/(2k)}) (1+\frac{\sigma^{1/k}(\log n)^{1/(2k)-1}}{2^{2-1/(2k)}k^{2}}(\log\log n-(k^{2}-1)\log2-2k\log\Gamma(1+k/2)) )}{ k (2^{1/(2k)}\sigma^{1/k}(\log n)^{1/(2k)}+\log (1+\frac{\sigma^{1/k}(\log n)^{1/(2k)-1}}{2^{2-1/(2k)}k^{2}}(\log\log n-(k^{2}-1)\log2-2k\log\Gamma(1+k/2)) ) )^{2k-1}}. \end{aligned}$$

It is easy to check that $\lim_{n\rightarrow\infty}\alpha_{n}/a_{n}=1$ and $\lim_{n\rightarrow\infty}(b_{n}-\beta_{n})/\alpha_{n}=0$. Hence, by Theorem 1.2.3 in Leadbetter *et al.* [16], the proof is complete. □

### Remark 3.2

Theorem 3.2 shows that the limit distribution of the normalized maximum from the logarithmic Maxwell distribution is the extreme value distribution $\exp(-\exp(-x))$ with norming constants

$$\alpha_{n}=\frac{\sigma^{2}\exp (2^{1/2}\sigma(\log n)^{1/2} ) (1+\frac{\sigma}{2^{3/2}(\log n)^{1/2}} (\log\log n-2\log(\pi^{1/2}/2) ) )}{ 2^{1/2}\sigma(\log n)^{1/2}+\log (1+\frac{\sigma }{2^{3/2}(\log n)^{1/2}} (\log\log n-2\log(\pi^{1/2}/2) ) )} $$

and

$$\beta_{n}=\exp \bigl(2^{1/2}\sigma(\log n)^{1/2} \bigr) \biggl(1+\frac {\sigma}{2^{3/2}(\log n)^{1/2}} \bigl(\log\log n-2\log\bigl(\pi ^{1/2}/2\bigr) \bigr) \biggr). $$

At the end of this section, we generalize the result of Theorem 3.2 to the situation of a finite blending of LGMDs.

Finite mixture distributions (or models) have been widely applied in various areas such as Chemistry [17] and image and video databases [18]. Specifically, related extreme statistical scholars have studied them. Mladenović [19] have considered extreme values of the sequences of independent random variables with common mixed distributions containing normal, Cauchy and uniform distributions. Peng *et al.* [20] have investigated the limit distribution and its corresponding uniform rate of convergence for a finite mixed of exponential distribution.

If the distribution function (df) *F* of a random variable *ξ* have

$$F(x)=p_{1}F_{1}(x)+p_{2}F_{2}(x)+ \cdots+p_{r}F_{r}(x), $$

we say that *ξ* obeys a finite mixed distribution *F*, where $F_{i}$, $1\leq i\leq r$ stand for different dfs of the mixture components. The weight coefficients satisfy the condition that $p_{i}>0$, $i=1,2,\ldots,r$ and $\sum^{r}_{j=1}p_{j}=1$.

Next, we think of the extreme value distribution from a finite blending with constituent dfs $F_{k_{i}}$ obeying $\operatorname{LGMD}(k_{i})$, where the parameter $k_{i}>1$ for $1\leq i\leq r$ and $k_{i}\neq k_{j}$ for $i\neq j$. Denote the cumulative df of the finite blending by

3.4 $$ F(x)=p_{1}F_{k_{1}}(x)+p_{2}F_{k_{2}}(x)+ \cdots+p_{r}F_{k_{r}}(x) $$

for $x>0$.

### Theorem 3.3

*Suppose that* $\{Z_{n},n\geq1\}$ *be a sequence of i*.*i*.*d*. *random variables following the common df* *F* *given by* (3.4). *Set* $M_{n}=\max\{Z_{k},1\leq k\leq n\}$. *Now*

$$\lim_{n\rightarrow\infty}P \biggl(\frac{M_{n}-\beta_{n}}{\alpha_{n}}\leq x \biggr)=\exp \bigl(-\exp(-x)\bigr) $$

*holds with the norming constants*

$$\alpha_{n}=\frac{\sigma^{1/k}\exp (2^{1/(2k)}\sigma^{1/k}(\log n)^{1/(2k)} )}{2^{1-1/(2k)}k(\log n)^{1-1/(2k)}} $$

*and*

$$\begin{aligned} \beta_{n} =&\exp \bigl(2^{1/(2k)}\sigma^{1/k}(\log n)^{1/(2k)} \bigr) \biggl(1+\frac{\sigma^{1/k}(\log n)^{1/(2k)-1}}{2^{2-1/(2k)}k^{2}} \bigl(\log\log n+2k\log p \\ &{}- \bigl(k^{2}-1\bigr)\log2-2k\log\Gamma(1+k/2) \bigr) \biggr), \end{aligned}$$

*where* $\sigma=\max\{\sigma_{1},\ldots,\sigma_{r}\}$, *and* $p=p_{i_{1}}+\cdots+p_{i_{j}}$, $i_{s}\in\{i,\sigma_{i}=\sigma\textit{ and }k=k_{i}\}$, $1\leq s\leq j\leq r$, *and* $k=\min\{k_{1},\ldots,k_{r}\}$.

### Proof

By (3.4), we have

$$1-F(x)=\sum^{r}_{i=1}p_{i} \bigl(1-F_{k_{i}}(x)\bigr). $$

By Theorem 2.1, we have

$$\begin{aligned}& \sum^{r}_{i=1}\frac{p_{i}\sigma^{2}_{i}}{k_{i}}(\log x)^{1-2k_{i}}xf_{k_{i}}(x) \\& \quad < 1-F(x)< \sum^{r}_{i=1} \frac{p_{i}\sigma^{2}_{i}}{k_{i}}(\log x)^{1-2k_{i}}x \biggl(1+\biggl(\frac{\sigma^{2}_{i}}{k_{i}}(\log x)^{2k_{i}}-1\biggr)^{-1} \biggr)f_{k_{i}}(x) \end{aligned}$$

for all $x>1$, according to the definition of $f_{k}$, which implies

3.5 $$\begin{aligned}& \frac{p\log x}{2^{\frac{k}{2}}\sigma^{\frac{1}{k}}\Gamma(1+\frac{k}{2})}\exp \biggl(-\frac{(\log x)^{2k}}{2\sigma^{2}} \biggr) \bigl(1+A_{k}(x)\bigr) \\& \quad < 1-F(x) \\& \quad < \frac{p\log x}{2^{\frac{k}{2}}\sigma^{\frac{1}{k}}\Gamma(1+\frac{k}{2})} \biggl(1+\biggl(\frac{\sigma^{2}}{k}(\log x)^{2k}-1\biggr)^{-1} \biggr)\exp \biggl(-\frac{(\log x)^{2k}}{2\sigma^{2}} \biggr) \bigl(1+B_{k}(x)\bigr), \end{aligned}$$

where

3.6 $$ A_{k}(x)=\sum_{k_{i}\neq k} \frac{2^{\frac{k}{2}}p_{i}\sigma^{\frac{1}{k}}\Gamma(1+\frac {k}{2})}{2^{\frac{k_{i}}{2}}p\sigma^{\frac{1}{k_{i}}}\Gamma(1+\frac{k_{i}}{2})} \exp \biggl(\frac{(\log x)^{2k}}{2\sigma^{2}}-\frac{(\log x)^{2k_{i}}}{2\sigma^{2}_{i}} \biggr) \rightarrow0 $$

and

3.7 $$\begin{aligned} B_{k}(x) =&\sum_{k_{i}\neq k} \frac{2^{\frac{k}{2}}p_{i}\sigma^{\frac{1}{k}}\Gamma(1+\frac {k}{2})}{2^{\frac{k_{i}}{2}}p\sigma^{\frac{1}{k_{i}}}\Gamma(1+\frac{k_{i}}{2})} \frac{1+(\frac{\sigma^{2}_{i}}{k_{i}}(\log x)^{2k_{i}}-1)^{-1}}{1+(\frac{\sigma^{2}}{k}(\log x)^{2k}-1)^{-1}} \exp \biggl(\frac{(\log x)^{2k}}{2\sigma^{2}}- \frac{(\log x)^{2k_{i}}}{2\sigma^{2}_{i}} \biggr) \\ \rightarrow&0 \end{aligned}$$

as $x\rightarrow\infty$ since $k=\min\{k_{1},k_{2},\ldots,k_{r}\}$. Combining (3.5)-(3.7) with (2.2), for large enough *x*, we obtain

3.8 $$ 1-F(x)\sim p\bigl(1-F_{k}(x)\bigr) $$

as $x\rightarrow\infty$, where $F_{k}$ represents the cdf of the $\operatorname{LGMD}(k)$, and *σ* and *p* are defined by Theorem 3.3. By Proposition 1.19 in Resnick [13], we can derive $F\in D(\Lambda)$. The norming constants can be obtained by Theorem 3.2 and (3.8). The desired result follows. □

## Asymptotic expansion of maximum

In this section, we establish an high-order expansion of the distribution of the extreme from the LGMD sample.

### Theorem 4.1

*For the norming constants* $a_{n}$ *and* $b_{n}$ *given by* (3.1), *we have*

$$ \lim_{n\rightarrow\infty}(\log b_{n})^{\lambda} \bigl((\log b_{n})^{2k-1}\bigl(F^{n}_{k}(a_{n}x+b_{n})- \Lambda(x)\bigr)-I(x)\Lambda(x) \bigr)=l(x)\Lambda(x), $$

*where*

$$\begin{aligned}& l(x)= \textstyle\begin{cases} J_{k}(x)+\frac{1}{2}I^{2}(x), &\textit{if } \frac{1}{2}< k< 1, \\ J_{k}(x)+w(x), &\textit{if } k=1, \\ J_{k}(x), &\textit{if } k>1, \end{cases}\displaystyle \\& I(x)=\frac{1}{2}k^{-1}\sigma^{2}x^{2}e^{-x}, \qquad w(x)=\frac{1}{4}\sigma ^{4}x^{4}e^{-2x}, \end{aligned}$$

*and*

$$ J_{k}(x)= \textstyle\begin{cases} k^{-2}\sigma^{4}x^{3} (\frac{1}{3}-\frac{1}{4}x )e^{-x}, &\textit{if } \frac{1}{2}< k< 1, \\ \sigma^{2}x (1+\frac{1}{2}x+\frac{1}{3}\sigma^{2}x^{2}-\frac {1}{4}\sigma^{2}x^{3} )e^{-x}, &\textit{if } k=1, \\ k^{-1}\sigma^{2}x (\frac{1}{2}(2k-1)x+1 )e^{-x}, &\textit{if } k>1. \end{cases} $$

### Corollary 4.1

*Under the condition of Theorem* 4.1, *we have*

$$ F^{n}_{k}(a_{n}x+b_{n})-\Lambda(x)\sim \frac{I(x)\Lambda(x)}{(2\sigma^{2}\log n)^{1-1/(2k)}} $$

*for large* *n*, *where* $I(x)$ *is given by Theorem* 4.1.

### Proof

The result directly follows from Theorem 4.1. The detailed proof is omitted. □

In order to prove Theorem 4.1, we need several lemmas. The following lemma shows a decomposition of the distributional tail representation of the LGMD.

### Lemma 4.1

*Let* $F_{k}(x)$ *denote the cdf of the LMGD*. *For large* *x*, *we have*

$$\begin{aligned} 1-F_{k}(x) =&\frac{1}{2^{k/2}\sigma^{1/k}\Gamma(1+k/2)}\exp \bigl(-1/\bigl(2 \sigma^{2}\bigr) \bigr) \bigl[1+k^{-1}\sigma^{2}( \log x)^{-2k} \\ &{}+k^{-2}(1-2k)\sigma^{4}(\log x)^{-4k}+O \bigl((\log x)^{-6k} \bigr) \bigr]\exp \biggl(- \int^{x}_{e}\frac{g(t)}{f(t)}\, \mathrm{dt} \biggr) \end{aligned}$$

*with* $f(t)$ *and* $g(t)$ *given by Theorem* 3.1.

### Proof

By integration by parts, we have

4.1 $$\begin{aligned} 1-F_{k}(x) =&\frac{k}{2^{\frac{1}{2}}\Gamma(1+\frac{k}{2})} \int ^{\infty}_{\log x/\sigma^{\frac{1}{k}}}s^{2k}\exp \biggl(- \frac{1}{2}s^{2k} \biggr)\, \mathrm{d}s \\ =&\frac{\log x}{2^{\frac{k}{2}}\sigma^{\frac{1}{k}}\Gamma(1+\frac{k}{2})}\exp \biggl(-\frac{(\log x)^{2k}}{2\sigma^{2}} \biggr) \bigl[1+k^{-1}\sigma^{2}(\log x)^{-2k} \\ &{}+k^{-2}(1-2k) \sigma^{4}(\log x)^{-4k}+k^{-3}(1-2k) (1-4k)\sigma^{6}(\log x)^{-6k} \bigr] \\ &{}+\frac{(1-2k)(1-4k)(1-6k)}{2^{\frac{k}{2}}k^{3}\Gamma(1+\frac {k}{2})} \int^{\infty}_{\log x/\sigma^{\frac{1}{k}}}s^{-6k}\exp \biggl(- \frac{1}{2}s^{2k} \biggr)\, \mathrm{d}s. \end{aligned}$$

Using L’Hospital’s rules yields

4.2 $$ \lim_{n\rightarrow\infty}\frac{\int^{\infty}_{\log x/\sigma^{\frac{1}{k}}} s^{-6k}\exp (-\frac{1}{2}s^{2k} )\,\mathrm{d}s}{(\log x)^{1-6k}\exp (-\frac{(\log x)^{2k}}{2\sigma^{2}} )}=0. $$

One easily checks that

4.3 $$\begin{aligned}& \frac{\log x}{2^{k/2}\sigma^{1/k}\Gamma(1+k/2)}\exp \biggl(-\frac{(\log x)^{2k}}{2\sigma^{2}} \biggr) \\& \quad =\frac{1}{2^{k/2}\sigma^{1/k}\Gamma(1+k/2)}\exp \biggl(-\frac {1}{2\sigma^{2}} \biggr)\exp \biggl(- \int^{x}_{e}\frac{g(t)}{f(t)}\, \mathrm{dt} \biggr) \end{aligned}$$

with $f(t)$ and $g(t)$ determined by Theorem 3.1. Combining with (4.1)-(4.3), we complete the proof. □

### Lemma 4.2

*Set*

$$ B_{n}(x) = \frac{1+k^{-1}\sigma^{2}(\log b_{n})^{-2k}+k^{-2}(1-2k)\sigma^{4}(\log b_{n})^{-4k}+O ((\log b_{n})^{-6k} )}{1+k^{-1}\sigma^{2}(\log(a_{n}x +b_{n}))^{-2k}+k^{-2}(1-2k)\sigma^{4}(\log(a_{n}x+b_{n}))^{-4k}+O ((\log (a_{n}x+b_{n}))^{-6k} )} $$

*with the norming constants* $a_{n}$ *and* $b_{n}$ *given by* (3.1), *then*

$$ B_{n}(x)-1=2k^{-1}\sigma^{4}(\log b_{n})^{-4k}x+O \bigl((\log b_{n})^{1-6k} \bigr). $$

### Proof

By (3.2), we have $n(1-F_{k}(a_{n}x+b_{n}))\rightarrow e^{-x}$ as $n\rightarrow\infty$, with the norming constants $a_{n}$ and $b_{n}$ given by (3.1). It is not difficult to verify that $\lim_{n\rightarrow\infty}B_{n}(x)=1$ and

4.4 $$\begin{aligned} B_{n}(x)-1 =& \bigl[k^{-1}\sigma^{2} \bigl((\log b_{n})^{-2k}-\bigl(\log(a_{n}x +b_{n})\bigr)^{-2k} \bigr) \\ &{}+k^{-2}(1-2k)\sigma^{4} \bigl((\log b_{n})^{-4k}- \bigl(\log(a_{n}x+b_{n})\bigr)^{-4k} \bigr) \\ &{}+O \bigl(( \log b_{n})^{-6k} \bigr) \bigr]\bigl(1+o(1)\bigr). \end{aligned}$$

For large *n* we have

4.5 $$\begin{aligned} (\log b_{n})^{-2k}-\bigl(\log(a_{n}x +b_{n})\bigr)^{-2k} =& 2\sigma^{2}(\log b_{n})^{-4k}x-k^{-1}\sigma^{4}(\log b_{n})^{1-6k}x^{2} \\ &{}+O \bigl((\log b_{n})^{2-8k} \bigr)+O \bigl((\log b_{n})^{-6k} \bigr) \end{aligned}$$

and

4.6 $$\begin{aligned} (\log b_{n})^{-4k}-\bigl(\log(a_{n}x +b_{n})\bigr)^{-4k} =& 4\sigma^{2}(\log b_{n})^{-6k}x-2k^{-1}\sigma^{4}(\log b_{n})^{1-8k}x^{2} \\ &{}+O \bigl((\log b_{n})^{2-10k} \bigr)+O \bigl((\log b_{n})^{-8k} \bigr). \end{aligned}$$

By (4.4)-(4.6), the desired result follows. □

### Lemma 4.3

*Set* $\lambda=1\wedge(2k-1)$ *to denote the minimum of* $\{1,2k-1\}$ *and* $v_{n}(x)=n\log F_{k}(a_{n}x+b_{n})+e^{-x}$ *with norming constants* $a_{n}$ *and* $b_{n}$ *given by* (3.1). *Then*

4.7 $$ \lim_{n\rightarrow\infty}(\log b_{n})^{\lambda} \bigl((\log b_{n})^{2k-1}v_{n}(x)-I(x) \bigr)=J_{k}(x), $$

*with* $I(x)$, $J_{k}(x)$ *given by Theorem* 4.1.

### Proof

For any positive integers *m* and $i>1$, by Corollary 2.1 and the fact that $1/(1-F_{k}(a_{n}x+b_{n}))=n$, we have

4.8 $$ \lim_{n\rightarrow\infty}\frac{(1-F_{k}(a_{n}x+b_{n}))^{i}}{n^{-1}(\log b_{n})^{-mk}}=0. $$

For any $x\in\mathbb{R}$ and $a_{n}=k^{-1}\sigma^{2}b_{n}(\log b_{n})^{1-2k}$, we have

4.9 $$ (\log b_{n})^{2k-1} \biggl(\frac{ka_{n}}{\sigma^{2}(a_{n}x+b_{n})(\log (a_{n}x+b_{n}))^{1-2k}}-1 \biggr)\rightarrow-k^{-1}\sigma^{2}x $$

and

4.10 $$ \frac{a_{n}(\log b_{n})^{2k-1}}{(a_{n}x+b_{n})\log(a_{n}x+b_{n})}\rightarrow0, $$

as $n\rightarrow\infty$. Here set

$$ C_{n}(x)=\frac{ka_{n}}{\sigma^{2}(a_{n}x+b_{n})(\log(a_{n}x+b_{n}))^{1-2k}}-\frac {a_{n}}{(a_{n}x+b_{n})\log(a_{n}x+b_{n})}-1. $$

By Lemmas 4.1, 4.2, (4.9), and (4.10), we have

4.11 $$\begin{aligned}& \frac{1-F_{k}(b_{n})}{1-F_{k}(a_{n}x+b_{n})}e^{-x} \\& \quad = B_{n}(x)\exp \biggl( \int^{x}_{0} \biggl(\frac{ka_{n}}{\sigma ^{2}(a_{n}s+b_{n})(\log(a_{n}s+b_{n}))^{1-2k}}- \frac{a_{n}}{(a_{n}s+b_{n})\log (a_{n}s+b_{n})}-1 \biggr)\,\mathrm{d}s \biggr) \\& \quad = B_{n}(x)\exp \biggl( \int^{x}_{0}C_{n}(s)\,\mathrm{d}s \biggr) \\& \quad = B_{n}(x) \biggl(1+ \int^{x}_{0}C_{n}(s)\,\mathrm{d}s+ \frac{1}{2} \biggl( \int ^{x}_{0}C_{n}(s)\,\mathrm{d}s \biggr)^{2}\bigl(1+o(1)\bigr) \biggr). \end{aligned}$$

By (4.8)-(4.11), Lemma 4.2, and the dominated convergence theorem, we have

4.12 $$\begin{aligned}& \lim_{n\rightarrow\infty}(\log b_{n})^{2k-1}v_{n}(x) \\& \quad = \lim_{n\rightarrow\infty}\frac{\log F_{k}(a_{n}x+b_{n})+n^{-1}e^{-x}}{n^{-1}(\log b_{n})^{1-2k}} \\& \quad = \lim_{n\rightarrow\infty}\frac{-(1- F_{k}(a_{n}x+b_{n}))-\frac{1}{2}(1- F_{k}(a_{n}x+b_{n}))^{2}(1+o(1))+(1-F_{k}(b_{n}))e^{-x}}{n^{-1}(\log b_{n})^{1-2k}} \\& \quad = \lim_{n\rightarrow\infty}\frac{1- F_{k}(a_{n}x+b_{n})}{n^{-1}}\frac{\frac {1-F_{k}(b_{n})}{1-F_{k}(a_{n}x+b_{n})}e^{-x}-1}{(\log b_{n})^{1-2k}} \\& \quad = e^{-x}\lim_{n\rightarrow\infty}(\log b_{n})^{2k-1} \biggl(B_{n}(x)+B_{n}(x) \int^{x}_{0}C_{n}(s)\,\mathrm{d}s \bigl(1+o(1)\bigr)-1 \biggr) \\& \quad = e^{-x}\lim_{n\rightarrow\infty}(\log b_{n})^{2k-1} \int^{x}_{0}C_{n}(s)\,\mathrm{d}s \\& \quad = -\frac{1}{2}k^{-1}\sigma^{2}x^{2}e^{-x} \\& \quad = :I(x). \end{aligned}$$

For all $x\in\mathbb{R}$,

$$\begin{aligned}& \frac{ka_{n}}{\sigma^{2}(a_{n}s+b_{n})(\log (a_{n}s+b_{n}))^{1-2k}}-1+k^{-1}\sigma^{2}s(\log b_{n})^{1-2k} \\& \quad = \bigl(1+k^{-1}\sigma^{2}(\log b_{n})^{1-2k}s \bigr)^{-1} \biggl((2k-1) \biggl(k^{-1}\sigma^{2}( \log b_{n})^{-2k}s-\frac{1}{2}k^{-2} \sigma^{4}(\log b_{n})^{1-4k}s^{2} \biggr) \\& \qquad {}+k^{-2}\sigma^{4}(\log b_{n})^{2-4k}s^{2}+O \bigl((\log b_{n})^{2-6k} \bigr) \biggr) \end{aligned}$$

for large *n*, which implies

4.13 $$\begin{aligned}& (\log b_{n})^{2k-1+\lambda} \biggl(\frac{ka_{n}}{\sigma^{2}(a_{n}s+b_{n})(\log (a_{n}s+b_{n}))^{1-2k}}-1+k^{-1} \sigma^{2}s(\log b_{n})^{1-2k} \biggr) \\& \quad \rightarrow \textstyle\begin{cases} k^{-2}\sigma^{4}s^{2}, &\mbox{if } \frac{1}{2}< k< 1, \\ \sigma^{2}s(1+\sigma^{2}s), &\mbox{if } k=1, \\(2k-1)k^{-1}\sigma^{2}s, &\mbox{if } k>1, \end{cases}\displaystyle \end{aligned}$$

and

4.14 $$ \frac{a_{n}(\log b_{n})^{2k-1+\lambda}}{(a_{n}s+b_{n})\log(a_{n}s+b_{n})} \rightarrow \textstyle\begin{cases} 0, &\mbox{if } \frac{1}{2}< k< 1, \\ \sigma^{2}, &\mbox{if } k=1, \\ k^{-1}\sigma^{2}, &\mbox{if } k>1, \end{cases} $$

as $n\rightarrow\infty$.

By (4.13), (4.14), and Lemma 4.2, we have

$$\begin{aligned}& \lim_{n\rightarrow\infty}(\log b_{n})^{\lambda} \bigl((\log b_{n})^{2k-1}v_{n}(x)-I(x) \bigr) \\& \quad = \lim_{n\rightarrow\infty}\frac{-(1- F_{k}(a_{n}x+b_{n}))+(1-F_{k}(b_{n}))e^{-x} (1-I(x)e^{x}(\log b_{n})^{1-2k} )}{n^{-1}(\log b_{n})^{1-2k-\lambda}} \\& \quad = \lim_{n\rightarrow\infty}\frac{1- F_{k}(a_{n}x+b_{n})}{n^{-1}}\frac{\frac {1-F_{k}(b_{n})}{1-F_{k}(a_{n}x+b_{n})}e^{-x} (1-I(x)e^{x}(\log b_{n})^{1-2k} )-1}{(\log b_{n})^{1-2k-\lambda}} \\& \quad = e^{-x}\lim_{n\rightarrow\infty} \biggl[(\log b_{n})^{2k+\lambda-1}\bigl(B_{n}(x)-1\bigr)+B_{n}(x) (\log b_{n})^{2k+\lambda-1} \\& \qquad {}\times \int^{x}_{0} \bigl(C_{n}(s)+k^{-1} \sigma^{2}s(\log b_{n})^{1-2k} \bigr)\,\mathrm{d}s -B_{n}(x)I(x)e^{x}(\log b_{n})^{\lambda} \int^{x}_{0}C_{n}(s)\,\mathrm{d}s \\& \qquad {}+\frac{1}{2}B_{n}(x) (\log b_{n})^{2k+\lambda-1} \bigl(1-I(x)e^{x}(\log b_{n})^{1-2k} \bigr) \biggl( \int^{x}_{0}C_{n}(s)\,\mathrm{d}s \biggr)^{2}\bigl(1+o(1)\bigr) \biggr] \\& \quad = \textstyle\begin{cases} k^{-2}\sigma^{4}x^{3} (\frac{1}{3}-\frac{1}{4}x )e^{-x},& \mbox{if } \frac{1}{2}< k< 1, \\ \sigma^{2}x (1+\frac{1}{2}x+\frac{1}{3}\sigma^{2}x^{2}-\frac {1}{4}\sigma^{2}x^{3} )e^{-x},& \mbox{if } k=1, \\ k^{-1}\sigma^{2}x (\frac{1}{2}(2k-1)x+1 )e^{-x},& \mbox{if } k>1 \end{cases}\displaystyle \\& \quad =: J_{k}(x), \end{aligned}$$

with $\lambda=1\wedge(2k-1)$. The proof is completed. □

### Proof of Theorem 4.1

By Lemma 4.3, we have

4.15 $$ (\log b_{n})^{2k-1+\lambda}v^{2}_{n}(x)\rightarrow \textstyle\begin{cases} I^{2}(x), &\mbox{if } \frac{1}{2}< k\leq1, \\ 0, &\mbox{if } k>1, \end{cases} $$

as $n\rightarrow\infty$. Once again by Lemma 4.3, we have

$$\begin{aligned}& (\log b_{n})^{\lambda} \bigl((\log b_{n})^{2k-1} \bigl(F^{n}_{k}(a_{n}x+b_{n})-\Lambda(x) \bigr)-I(x)\Lambda(x) \bigr) \\& \quad = (\log b_{n})^{\lambda} \bigl((\log b_{n})^{2k-1} \bigl(\exp\bigl(u_{n}(x)\bigr)-1\bigr)-I(x) \bigr)\Lambda(x) \\& \quad = \biggl((\log b_{n})^{\lambda} \bigl((\log b_{n})^{2k-1}v_{n}(x)-I(x) \bigr)+(\log b_{n})^{2k+\lambda-1}v^{2}_{n}(x) \biggl( \frac{1}{2}+O\bigl(v_{n}(x)\bigr) \biggr) \biggr)\Lambda(x) \\& \quad \rightarrow l(x)\Lambda(x), \end{aligned}$$

where $l(x)$ is provided by Theorem 4.1. The proof is completed. □

## Conclusion

Motivated by Vodă [1], we put forward the logarithmic generalized Maxwell distribution. We discuss tail properties and the limit distribution of the distribution. We extend the results to the case of a finite mixture distribution. With the optimal norming constants, we establish the high-order expansion of the distribution of maxima from logarithmic generalized Maxwell distribution, by which we derive the convergence rate of the distribution of maximum to the associate extreme limit.
